# Sensibilidade e Especificidade de Pontos de Corte para Frequência Cardíaca em Repouso de 6.794 Adolescentes Brasileiros: Um Estudo Transversal

**DOI:** 10.36660/abc.20200111

**Published:** 2021-07-15

**Authors:** Breno Quintella Farah, Diego Giulliano Destro Christofaro, Aluísio Andrade-Lima, Antonio Henrique Germano-Soares, William Rodrigues Tebar, Mauro Virgílio Gomes de Barros, Raphael Mendes Ritti-Dias

**Affiliations:** 1 Programa de Pós-Graduação em Educação FísicaUniversidade Federal de PernambucoRecifePEBrasilPrograma de Pós-Graduação em Educação Física da Universidade Federal de Pernambuco, Recife, PE - Brasil; 2 Universidade Estadual PaulistaSão PauloSPBrasilUniversidade Estadual Paulista (UNESP), São Paulo, SP - Brasil; 3 Universidade Federal de SergipeAracajuSEBrasilUniversidade Federal de Sergipe, Aracaju, SE - Brasil; 4 Programa Associado de Pós-Graduação em Educação FísicaUPE/UFPBRecifePEBrasilPrograma Associado de Pós-Graduação em Educação Física UPE/UFPB, Recife, PE - Brasil; 5 Universidade Nove de JulhoPrograma de Pós-Graduação em Ciências da ReabilitaçãoSão PauloSPBrasilUniversidade Nove de Julho - Programa de Pós-Graduação em Ciências da Reabilitação, São Paulo, SP - Brasil

**Keywords:** Frequência Cardíaca, Adolescente, Epidemiologia, Adiposidade, Pressão Arterial, Fatores de Risco, Obesidade, Estudos Transversais

## Abstract

**Fundamento:**

A frequência cardíaca em repouso (FCR) pode ser uma ferramenta útil de triagem para o risco cardiovascular. Porém, os pontos de corte para FCR nunca foram descritos em populações jovens.

**Objetivo:**

Estabelecer os pontos de corte para FCR em adolescentes brasileiros e analisar se há associação entre pontos de corte e fatores de risco cardiovascular.

**Métodos:**

A amostra foi composta por 6.794 adolescentes (de 10 a 19 anos). A pressão arterial e a FCR foram avaliadas por dispositivo oscilométrico. Também foram avaliados o índice de massa corporal e a circunferência da cintura. Foi adotada a curva ROC para analisar a sensibilidade e especificidade, e as associações de FCR elevada com os fatores de risco cardiovascular foram analisadas por regressão logística binária. Foi considerado estatisticamente significante um valor de p < 0,05 para todas as análises.

**Resultados:**

Os valores médios da FCR eram mais altos entre os participantes de 10 a 14 anos do naqueles de 15 a 19 anos, em meninos (p < 0,001) e meninas (< 0,001). Os pontos de corte de FCR propostos para detecção de fatores de risco cardiovascular foram significativos para meninos de 10 a 14 (> 92 bpm) e de 15 a 19 anos (> 82 bpm) e para meninas de 15 a 19 anos (> 82 bpm) (p < 0,05 para todos), enquanto nenhum ponto de corte foi identificado para as meninas de 10 a 14 anos (p > 0,05). Os pontos de corte propostos para a FCR foram associados com obesidade abdominal, sobrepeso e pressão arterial elevada em meninos e meninas. Os pontos de corte da FCR foram associados ao conjunto de fatores de risco cardiovascular em adolescentes de 15 a 19 anos.

**Conclusões:**

Os pontes de corte propostos para a FCR foram associados com os fatores de risco cardiovascular em adolescentes.

## Introdução

A frequência cardíaca em repouso (FCR) é uma medida acessível que reflete o equilíbrio entre o sistema nervoso simpático e parassimpático.^1^ A FCR elevada tem sido associada a eventos cardiovasculares adversos e à mortalidade em adultos.^2,3^ Tem sido associada também à pressão arterial elevada, à obesidade abdominal e ao sobrepeso^4-8^ em crianças e adolescentes.^8^ A FCR elevada na infância e na adolescência parece rastrear o risco de doença cardiovascular até a idade adulta.^9^ Portanto, essa medida pode ser uma ferramenta útil de triagem de risco cardiovascular em populações jovens.

Em crianças e adolescentes, a FCR diminui com a idade e é dependente do sexo.^10-12^ Devido a isso, estudos realizados pelo *National Health and Nutrition Examination Survey* (NHANES 1999-2008) nos Estados Unidos,^12^ um estudo internacional,^13^ e a *German Health Interview and Examination Survey on Children and Adolescents *(KiGGS) têm apresentado percentis para FCR por idade e sexo, para crianças e jovens com idades de 3 a 17 anos.^5^

Apesar dos percentis fornecidos por estudos anteriores, nunca foram descritos os pontos de corte para FCR, o que seria uma abordagem clínica mais interessante para melhorar a utilidade desse marcador.^14-16^ Além disso, quase todos os estudos foram realizados em países de alta renda,^5,12,13^ e não se sabe se tais resultados poderiam ser extrapolados para adolescentes que vivem em países de renda baixa a média (por exemplo, o Brasil). Desta maneira, o objetivo principal deste estudo foi o de estabelecer os pontos de corte da FCR em adolescentes de 10 a 19 anos. A análise secundária visou explorar se esses pontos de corte estavam associados a fatores de risco cardiovascular.

## Métodos

### Desenho e amostra do estudo

Este estudo transversal foi realizado a partir das bases de dados de três estudos de base escolar das seguintes populações: 1) alunos de 14 a 19 anos da rede pública de ensino do estado de Pernambuco (Região Nordeste);^4^ 2) alunos de 10 a 17 anos da rede pública de ensino da cidade de Londrina (Paraná, Região Sul);^17^ e um estudo envolvendo alunos de 10 a 17 anos de escolas públicas e privadas da cidade de Presidente Prudente (São Paulo, Região Sudeste).^18^ Portanto, a população-alvo do presente estudo foram alunos de 10 a 19 anos de idade. Os três estudos foram aprovados pelos comitês de ética da Universidade de Pernambuco, da Universidade Estadual de Londrina e da Universidade Estadual Paulista, de acordo com as Diretrizes do Sistema Nacional de Ética em Pesquisa. Os procedimentos dos estudos foram descritos anteriormente.^4,17^

Ambos os estudos tinham critérios de inclusão semelhantes, tais como ausência de diabetes mellitus, doença cardiovascular ou deficiência neurológica ou mental conhecidos. Para as medidas cardiovasculares (FCR e pressão arterial), os adolescentes foram excluídos se relatassem qualquer um dos seguintes fatores: uso de álcool, uso de qualquer forma de tabaco e/ou drogas ilícitas ou realização de exercício físico no dia da medição.

Não foram consideradas variáveis comportamentais como nível de atividade física e comportamento não saudável no presente estudo devido às diferenças metodológicas nos instrumentos utilizados entre os estudos. Portanto, no presente estudo, foram compiladas apenas variáveis biológicas como sexo, idade, pressão arterial, FCR e medidas antropométricas (peso, altura e circunferência da cintura) que foram obtidas utilizando procedimentos semelhantes.

### Frequência cardíaca em repouso - Preditores

Foram obtidas as medições da FCR utilizando o dispositivo de monitoramento de pressão arterial Omron HEM 742 (Omron, Xangai, China), que foi previamente validado.^19^ Após aproximadamente 30 minutos de repouso (tempo aproximado para responder ao questionário) e um período de pelo menos cinco minutos na posição sentada, todos os adolescentes tiveram sua FCR medida três vezes, sendo as duas últimas medidas consideradas para análise.

### Desfechos

#### Pressão Arterial

A pressão arterial foi medida com o dispositivo Omron HEM 742 (Omron, Xangai, China).^19^ Cada adolescente permaneceu na posição sentada durante pelo menos cinco minutos, com as pernas descruzadas. A pressão arterial foi medida no braço direito, com manguito de tamanho adequado posicionado na altura do coração. Para análise, foram consideradas as médias das últimas duas medidas, e os adolescentes foram classificados como tendo pressão arterial elevada quando a pressão arterial sistólica e/ou diastólica foi igual ou maior que o percentil 95 de referência para sexo, idade e altura.^20^

#### Sobrepeso e obesidade abdominal

A altura e o peso foram obtidos com os adolescentes descalços e sem casacos por meio de estadiômetro e balança automática, respectivamente. O sobrepeso foi determinado conforme proposto por Cole et al.^21^ A circunferência da cintura foi obtida na posição ortostática na altura do umbigo utilizando uma fita de tensão constante, e os adolescentes com valores de circunferência da cintura acima do percentil 80 referente para sexo e idade foram considerados com obesidade abdominal.^22^

#### Conjunto de fatores de risco cardiovascular

O agrupamento dos fatores de risco cardiovascular foi determinado pela soma de obesidade abdominal, sobrepeso e pressão arterial elevada. Para análise, foram incluídos apenas adolescentes com todas as informações, e a pontuação do conjunto variou de 0 a 3.

#### Análise estatística

Os três bancos de dados foram compilados por um único pesquisador em uma planilha de dados usando o software SPSS/PASW (IBM Corp, New York, EUA) e os possíveis erros foram verificados por um segundo pesquisador independente. A distribuição normal foi analisada por análise gráfica (histograma). As variáveis contínuas foram resumidas como médias, desvio padrão e percentis, enquanto as variáveis categóricas foram resumidas como frequência relativa. Foram realizadas as comparações de idade e FCR entre os sexos usando o teste t independente, enquanto o teste do qui-quadrado foi utilizado para comparar as variáveis categóricas (obesidade abdominal, sobrepeso, pressão arterial elevada e o conjunto de fatores de risco cardiovascular).

A sensibilidade e especificidade da FCR na detecção de fatores de risco cardiovascular foram realizadas usando a curva de ROC. A associação da FCR elevada e os fatores de risco cardiovascular separados ou em conjunto foi realizada pela regressão logística binária. O intervalo de confiança adotado foi de 95% e a significância estatística foi de 5%. Os pacotes estatísticos usados foram SPSS e Medcalc.

## Resultados

O número de adolescentes elegíveis, avaliados e excluídos foi descrito anteriormente.^4,17,18^ Desta maneira, foram incluídos no estudo 6.794 adolescentes (4.040 meninas e 2.754 meninos) com média de idade de 16,0 ± 1,8 anos. As características dos adolescentes são apresentadas na Tabela 1. As meninas apresentaram maior FCR e obesidade abdominal, enquanto os meninos apresentaram maior prevalência de pressão arterial elevada.

**Tabela 1:** – Características gerais de adolescentes brasileiros de acordo com sexo, em 2011 (n = 6.794)

	Total	Meninos (n=2.754)	Meninas (n=4.040)	p
Idade (anos)	16,0 ± 1,8	16,0 ± 1,9	16,0 ± 1,7	0,427^a^
Frequência cardíaca em repouso (bpm)	78,4 ± 12,8	74,4 ± 12,6	81,1 ± 12,2	<0,001^a^
Obesidade abdominal (%)	20,7	16,5	23,5	<0,001^b^
Sobrepeso (%)	17,9	18,0	17,8	0,842^ b^
Pressão arterial elevada (%)	16,6	24,0	11,6	<0,001^ b ^
**Fatores de risco (%)**				**<0,001**^**b**^
Nenhum	66,1	64,5	67,2	<0,001^ b^
Um	17,6	19,0	16,6	
Dois	11,3	9,9	12,3	
Três	5,0	6,6	3,9	

*bpm: batimentos por minuto; ^a^: t teste independente; ^b^: teste do qui-quadrado.*

**Figura 1:**
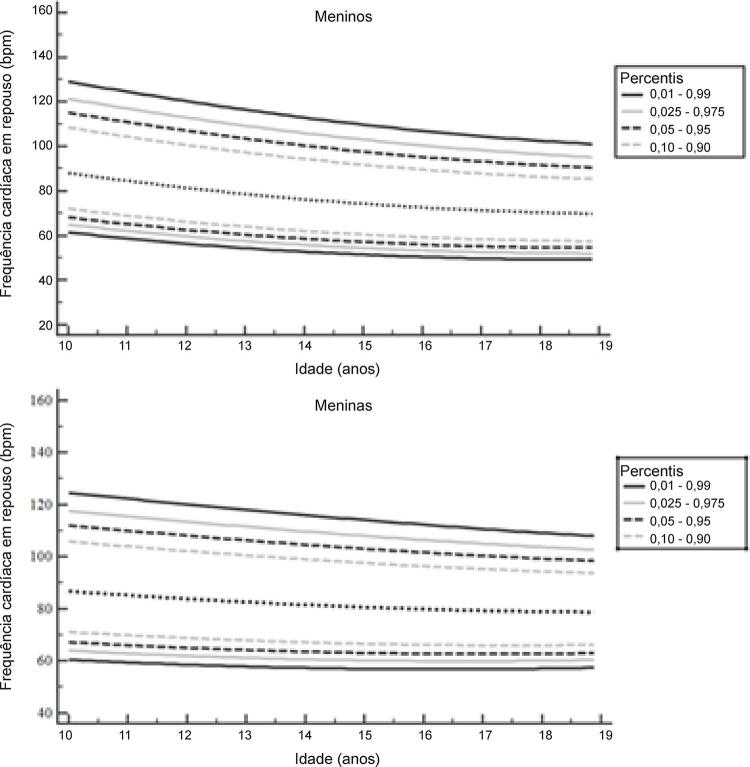
– Frequência cardíaca em repouso suavizada para adolescentes brasileiros de sexo masculino e feminino (n = 6.794). bpm: batimentos por minuto.

Os percentis da FCR de adolescentes por idade e sexo são mostrados na Figura 1 e na Tabela 2. A FCR média para meninos de 10 a 14 anos foi maior do que em meninos de 15 a 19 anos (80,7 ± 13,0 bpm versus 73,0 ± 12,0 bpm, p < 0,001). A FCR média para meninas de 10 a 14 anos também foi maior do que para meninas de 15 a 19 anos (83,8 ± 13,0 bpm versus 80,6 ± 12,0 bpm, p < 0,001).

**Tabela 2:** – Médio ± desvio padrão e valores dos percentis para frequência cardíaca em repouso em adolescentes de 10 a 19 anos de idade

	Idade (anos)	Médio ± DP	Percentis para frequência cardíaca em repouso								
			1	2,5	5	10	50	90	95	97,5	99
**Meninos (n=2.754)**	10	85,3 ± 14,6	61,4	64,9	68,1	72,1	83,0	108,4	115,2	121,4	129,1
	11	85,7 ± 12,6	58,7	62,1	65,2	69,0	87,5	104,4	110,9	117,0	124,5
	12	84,4 ± 13,6	56,3	59,6	62,6	66,3	83,5	100,7	107,1	113,0	120,3
	13	80,7 ± 11,3	54,3	57,5	60,4	64,0	79,0	97,3	103,5	109,3	116,4
	14	77,8 ± 12,3	52,7	55,8	58,6	62,1	77,8	94,4	100,4	106,0	112,9
	15	76,1 ± 11,6	51,4	54,4	57,1	60,5	75,0	91,8	97,7	103,1	109,8
	16	73,4 ± 12,5	50,4	53,3	56,0	59,2	73,4	89,6	95,3	100,5	107,0
	17	72,1 ± 12,0	49,7	52,5	55,1	58,3	71,0	87,8	93,3	98,3	104,6
	18	70,7 ± 11,5	49,3	52,1	56,6	57,7	70,0	86,4	91,6	96,5	102,5
	19	72,3 ± 10,9	49,8	54,6	56,0	60,0	70,0	86,0	90,0	99,4	111,6
**Meninas (n=4.040)**	10	85,9 ± 11,5	61,3	64,5	67,6	71,3	86,0	106,2	112,7	118,8	126,5
	11	86,4 ± 12,4	60,2	63,4	66,4	70,0	84,0	104,2	110,7	116,7	124,1
	12	87,0 ± 13,6	59,3	62,5	65,4	68,9	85,5	102,4	108,7	114,6	121,9
	13	83,4 ± 14,8	58,6	61,7	64,6	68,0	81,0	100,8	106,9	112,6	119,7
	14	82,1 ± 12,0	58,1	61,1	63,9	67,3	81,0	99,2	105,2	110,7	117,6
	15	82,2 ± 12,9	57,7	60,7	63,4	66,8	81,0	97,8	103,6	109,0	115,6
	16	80,1 ± 11,4	57,5	60,4	63,1	66,4	79,0	96,6	102,2	107,3	113,8
	17	80,5 ± 12,1	57,5	60,4	63,0	66,2	79,0	95,5	100,9	105,8	112,0
	18	79,4 ± 11,5	57,7	60,5	63,0	66,2	78,0	94,5	99,7	104,5	110,4
	19	80,1 ± 11,5	54,0	59,3	63,6	67,0	79,0	95,9	102,5	107,0	112,7

*DP: desvio padrão.*

Os pontos de corte propostos para a detecção de FCR elevada em ambos os sexos de acordo com a idade são apresentados na Tabela 3. Para meninas de 10 a 14 anos, a área sob a curva não foi significante no ponto de corte encontrado (p > 0,05), enquanto para meninos (de 10 a 14 e de 15 a 19 anos) e meninas de 15 a 19 anos, a área sob a curva foi significante (p < 0,05).

**Tabela 3:** – Pontos de corte propostos e indicadores da curva de característica de operação do receptor de detecção de frequência cardíaca em repouso para fatores de risco cardiovascular em adolescentes (n = 6.773)

	Corte	AUC	IC 95%	p	Sensibilidade	IC 95%	Especificidade	IC 95%
Meninos 10 a 14 anos	>92	0,632	0,588 – 0,675	0,021	44,44	25,5 – 64,7	81,00	77,1 – 84,5
Meninos 15 a 19 anos	>82	0,633	0,612 – 0,653	<0,001	35,96	26,1 – 46,8	82,67	81,0 – 84,2
Meninas 10 a 14 anos	>94	0,528	0,486 – 0,570	0,635	32,14	15,9 – 52,4	80,78	77,2 – 84,0
Meninas 15 a 19 anos	>82	0,709	0,693 – 0,724	<0,001	75,86	65,5 – 84,4	58,43	56,7 – 60,1

*AUC: área sob a curva; FCR: frequência cardíaca em repouso; IC: intervalo de confiança.*

A Tabela 4 mostra as associações entre os pontos de corte da FCR e os fatores de risco cardiovascular. Em meninos de 10 a 14 anos, a FCR elevada foi associada com a obesidade abdominal e o sobrepeso. Em meninos de 15 a 19 anos, a FCR elevada foi associada com a obesidade abdominal, o sobrepeso e a pressão arterial elevada. Em meninas de 15 a 19 anos, a FCR elevada foi associada com a obesidade abdominal, o sobrepeso e a pressão arterial elevada. Além disso, em meninos e meninas (de 15 a 19 anos), os pontos de corte da FCR foram associados ao conjunto de fatores de risco cardiovascular (Tabela 5).

**Tabela 4:** – Análises brutas e ajustadas da associação entre pontos de corte para frequência cardíaca em repouso e fatores de risco cardiovascular em adolescentes

Variáveis independentes	Modelos	FCR elevada^a^	
		RC (IC 95%)	
**Meninos (10 a 14 anos)**			
Obesidade abdominal (não = referência)	Bruto	2,46 (1,60 – 3,79)	
	Ajustado	2,37 (1,53 – 3,68)	
Sobrepeso (não = referência)	Bruto	2,06 (1,38 – 3,10)	
	Ajustado	1,87 (1,23 – 2,83)	
Pressão arterial elevada (não = referência)	Bruto	1,45 (0,93 – 2,26)	
	Ajustado	1,42 (0,90 – 2,23)	
**Meninos (15 a 19 anos)**			
Obesidade abdominal (não = referência)	Bruto	1,89 (1,27 – 2,82)	
	Ajustado	1,85 (1,24 – 2,76)	
Sobrepeso (não = referência)	Bruto	1,88 (1,27 – 2,78)	
	Ajustado	1,89 (1,28 – 2,80)	
Pressão arterial elevada (não = referência)	Bruto	2,83 (2,01 – 3,98)	
	Ajustado	3,00 (2,12 – 4,23)	
**Meninas (15 a 19 anos)**			
Obesidade abdominal (não = referência)	Bruto	1,22 (1,04 – 1,43)	
	Ajustado	1,26 (1,07 - 1,47)	
Sobrepeso (não = referência)	Bruto	1,26 (1,05 – 1,50)	
	Ajustado	1,27 (1,06 – 1,51)	
Pressão arterial elevada (não = referência)	Bruto	2,86 (2,28 – 3,59)	
	Ajustado	2,82 (2,25 – 3,54)	

*Ajustado por idade, período do dia. FCR: frequência cardíaca em repouso; IC: intervalo de confiança; RC: razão de chances. ^a^ FCR elevada: meninos de 10 a 14 anos: > 83 bpm; meninos de 15 a 19 anos: > 92 bpm; meninas de 10 a 14 anos: não disponível; meninas de 15 a 19 anos: > 82 bpm.*

**Tabela 5:** – Análises ajustadas da associação entre pontos de corte para frequência cardíaca em repouso e agrupamento de fatores de risco cardiovascular em conjuntos em adolescentes

Fatores de risco	FCR elevada^a^
	RC (IC 95%)
**Meninos (10 a 14 anos)**	
Nenhum	Referência
Um	0,86 (0,50 – 1,49)
Dois	2,48 (1,43 – 4,28)
Três	2,33 (1,20 – 4,52)
**Meninos (15 a 19 anos)**	
Nenhum	Referência
Um	3,14 (2,12 – 4,67)
Dois	2,22 (1,26 – 3,90)
Três	3,72 (2,12 – 6,54)
**Meninas (15 a 19 anos)**	
Nenhum	Referência
Um	1,33 (1,11 – 1,60)
Dois	1,24 (1,01 – 1,54)
Três	3,70 (2,47 – 5,53)

*Ajustado por idade, período do dia. FCR: frequência cardíaca em repouso; IC: intervalo de confiança; RC: razão de chances. ^a^ FCR elevada: meninos de 10 a 14 anos: > 83 bpm; meninos de 15 a 19 anos: > 92 bpm; meninas de 10 a 14 anos: não disponível; meninas de 15 a 19 anos: > 82 bpm.*

## Discussão

Os principais achados do presente estudo foram: a) os pontos de corte da FCR apresentam alta especificidade para a detecção de risco cardiovascular em meninos de 10 a 14 e de 15 a 19 anos; b) em meninas de 15 a 19 anos, os pontos de corte da FCR têm alta sensibilidade na detecção de risco cardiovascular, enquanto o ponto de corte não pôde ser estabelecido em meninas de 10 a 14 anos; c) os pontos de corte da FCR identificados foram associados ao conjunto de fatores de risco cardiovascular.

Neste estudo, houve declínio da FCR com o aumento da idade, de modo semelhante a outros estudos.^5,12^ Isto pode ser explicado pela melhoria da sensibilidade barorreflexa e da função neural com a maturação sexual.^23^ De fato, durante a maturação ocorre um aumento progressivo da atividade cardíaca parassimpática em relação a atividade simpática,^23^ resultando em menor FCR no final da adolescência.^24^

A FCR diferiu entre meninos e meninas de 15 a 19 anos (meninos: 73,0 ± 12,0 bpm versus meninas: 80,6 ± 12,0 bpm), corroborando com estudos anteriores.^4,5^ Tais diferenças podem ser causadas pela maior adiposidade nas meninas.^25^ Estudos anteriores também mostraram que tanto a aptidão cardiorrespiratória quanto os níveis de atividade física são mais elevados em meninos do que em meninas,^26^ e estes fatores estão diretamente relacionados ao controle autonômico cardíaco.^27-29^

Estudos anteriores reportaram valores de referência da FCR em crianças e adolescentes do nascimento aos 18 anos de idade, incluindo uma revisão sistemática envolvendo 143.346 participantes, que mostrou valores medianos de 123 bpm em adolescentes de 12 a 18 anos.^13^ Outros estudos que analisaram grandes populações nacionais, como o KiGGS^5^ e o NHANES,^12^ observaram valores medianos da FCR, em toda a faixa etária, variando entre 69 e 104 bpm para meninos e 74 e 108 bpm para meninas. Em comparação com esses estudos, observamos uma FCR mais homogênea (valores medianos de 70 a 83 bpm para meninos e 79 a 86 bpm para meninas), o que pode ser explicado pelo menor amplitude de faixa etária incluída no estudo.

A principal novidade do presente estudo foi a identificação de pontos de corte para a FCR em adolescentes. Tais pontos foram associados a importantes fatores de risco cardiovascular, tais como, a obesidade abdominal, o sobrepeso e a pressão arterial elevada, condições diretamente relacionadas com o controle autonômico cardíaco. Isso Explica, pelo menos em parte, a associação desses fatores com a FCR. Da mesma forma, os pontos de corte da FCR também foram associados ao conjunto de fatores de risco cardiovascular independentemente do sexo, indicando que o acúmulo de fatores de risco leva a maiores alterações na função autonômica cardíaca.

Os principais pontos fortes do presente estudo são o tamanho da amostra relativamente grande, a inclusão de uma ampla faixa etária, o uso de uma técnica automatizada para medições da FCR para evitar viés do observador e o fato de que a análise de dados foi realizada por um único pesquisador de maneira cega. Ademais, adolescentes com condiçoes ou medicamentos que influenciam a FCR foram excluídos. Apesar destes pontos fortes, o estudo presente apresenta algumas limitações que precisam ser consideradas. Primeiro, tivemos apenas uma única avaliação de FCR. Não foi possível determinar o estágio maturacional. Portanto, estudos futuros deverão considerar a maturação sexual dos adolescentes. O delineamento transversal deste estudo constitui uma limitação, pois não se pode inferir causalidade, o que torna necessário estudos longitudinais para validar os pontos de corte encontrados. Embora este seja o primeiro estudo com pontos de corte em adolescentes brasileiros, analisamos poucas regiões do Brasil, sendo necessários mais estudos multicêntricos. Por último, como outros potenciais confundidores que podem afetar a FCR (tabagismo, consumo de álcool e exercício antes da medição) não foram controlados, não se pode desconsiderar a sua influência. Apesar disso, estudos anteriores também adotaram uma estratégia semelhante.^5,12,13^

## Conclusão

O presente estudo identificou como pontos de corte para a FCR > 92 bpm para meninos de 10 a 14 anos, > 82 bpm para meninos de 15 a 19 anos e > 82 para meninas de 15 a 19 anos, e demonstrou uma associação entre estes pontos de corte e o conjunto de fatores de risco cardiovascular. Estes pontos de corte podem auxiliar médicos e outros profissionais de saúde a interpretar e classificar o risco cardiovascular, utilizando uma medida simples, fácil e de baixo custo.
