# Supplementary material for: Sensibilidade e Especificidade de Pontos de Corte para Frequência Cardíaca em Repouso de 6.794 Adolescentes Brasileiros: Um Estudo Transversal
Source: Arq Bras Cardiol. 2021 Jul 15;117(1):74–81. [Article in Portuguese] doi: 10.36660/abc.20200111 (PMC8294731; doi:10.36660/abc.20200111)
Supplement: Translation

# Translation: Sensitivity and Specificity of Cutoff Points of Resting Heart Rate from 6,794 Brazilian Adolescents: A Cross-Sectional Study

**Authors:** Breno Quintella Farah, Diego Giulliano Destro Christofaro, Aluísio Andrade-Lima, Antonio Henrique Germano-Soares, William Rodrigues Tebar, Mauro Virgílio Gomes de Barros, Raphael Mendes Ritti-Dias

**Affiliations:** 1 BrasilPrograma de Pós-Graduação em Educação Física da Universidade Federal de Pernambuco, Recife, PE - Brasil; 2 BrasilUniversidade Estadual Paulista (UNESP), São Paulo, SP - Brasil; 3 BrasilUniversidade Federal de Sergipe, Aracaju, SE - Brasil; 4 BrasilPrograma Associado de Pós-Graduação em Educação Física UPE/UFPB, Recife, PE - Brasil; 5 BrasilUniversidade Nove de Julho - Programa de Pós-Graduação em Ciências da Reabilitação, São Paulo, SP - Brasil

**Keywords:** Heart Rate, Adolescent, Epidemiology, Adiposity, Blood Arterial, Risk Factors, Obesity, Cross-Sectional Studies

## Abstract

**Background:**

Resting heart rate (RHR) may be a useful screening tool for cardiovascular risk. However, RHR cutoff points, an interesting clinical approach, have never been described in young populations.

**Objective:**

To establish RHR cutoff points in Brazilian adolescents and to analyze whether cutoff points are associated with cardiovascular risk factors.

**Methods:**

The sample was composed of 6,794 adolescents (10 to 19 years old). Blood pressure and RHR were assessed by oscillometric device. Body mass index and waist circumference were also assessed. Receiver operating characteristic curve was adopted to analyze the sensitivity and specificity, and associations of high RHR with cardiovascular risk factors were analyzed by binary logistic regression. A p value < 0.05 was considered statistically significant for all the analyses.

**Results:**

Mean RHR values were higher among participants ages 10 to 14 years than 15 to 19 years, for boys (p < 0.001) and girls (< 0.001). The proposed RHR cutoff points for cardiovascular risk factors detection were significant for boys ages 10 to 14 (> 92 bpm) and 15 to 19 years (> 82 bpm), as well as for girls ages 15 to 19 years (> 82 bpm) (p < 0.05 for all), whereas no cutoff point was identified for girls ages 10 to 14 years (p > 0.05). Proposed RHR cutoff points were associated with abdominal obesity, overweight, and high blood pressure in boys in girls. RHR cutoff points were associated with the cluster of cardiovascular risk factors in adolescents ages 15 to 19 years.

**Conclusion:**

The proposed RHR cutoff points were associated with cardiovascular risk factors in adolescents.

## Introduction

Resting heart rate (RHR) is an accessible measure that reflects the balance between the sympathetic and parasympathetic nervous systems.^1^ Elevated RHR has been associated with adverse cardiovascular disease events and mortality in adults.^2,3^ It has also been associated with high blood pressure, abdominal obesity, and overweight,^4-8^ including in children and adolescents.^8^ Elevated RHR in childhood and adolescence seems to track long-term risk of cardiovascular disease to adulthood.^9^ Therefore, RHR may be a useful screening tool for cardiovascular risk in young populations.

In children and adolescents, RHR decreases with age and is sex dependent.^10-12^ Thus, RHR percentiles derived in the National Health and Nutrition Examination Survey (NHANES 1999-2008) in the United States,^12^ an international study,^13^ and the German Health Interview and Examination Survey on Children and Adolescents (KiGGS) have presented RHR percentiles by age and sex, from the ages of 3 to 17 years.^5^

Despite the percentiles provided in previous studies, RHR cutoff points, which would be a more interesting clinical approach to improve the usefulness of this marker,^14-16^ have never been described. Likewise, as almost all of the studies were conducted in high-income countries,^5,12,13^ it is still unknown whether such results can be extrapolated for adolescents living in low- to middle-income countries (e.g., Brazil). Thus, the primary purpose of this study was to establish RHR cutoff points in 10 to 19 year olds. Secondary analysis aimed to explore whether these critical values were associated with cardiovascular risk factors.

## Methods

### Study design and sample

This cross-sectional study was performed using the databases from three school-based studies in the following populations: 1) students ages 14 to 19 years from the public school system in the state of Pernambuco (Northeast Region of Brazil)^4^; 2) students ages 10 to 17 years from the public school system in the city of Londrina (state of Paraná, South Region of Brazil);^17^ and students ages 10 to 17 years from both public and private schools in the city of Presidente Prudente (state of São Paulo, Southeast Region of Brazil).^18^ Therefore, the target population in the current study was students ages 10 to 19 years old. The three studies were approved by the ethics committees of the University of Pernambuco, Londrina State University, and São Paulo State University, in compliance with the Brazilian National Research Ethics System Guidelines. The study procedures have been previously described.^4,17^

Both studies had similar inclusion criteria, such as without known diabetes mellitus, cardiovascular disease, or neurological or mental disabilities. Due to cardiovascular measures (RHR and blood pressure), adolescents were excluded if they reported any of the following: alcohol use, use of any form of tobacco and/or illicit drugs, and participation in any physical exercise training on the day of measurement.

Behavioral variables such as physical activity level and unhealthy behavior were not considered in the present study due to methodological differences in the instruments used among studies. Therefore, only biological variables such as sex, age, blood pressure, RHR, and anthropometric measurements (weight, height, and waist circumference) with similar procedures were compiled in the current study.

### Resting heart rate - Predictors

RHR measurements were obtained using the Omron HEM 742 blood pressure monitoring device (Omron, Shangai, China), which has been previously validated ^19^. After approximately 30 minutes at rest (approximate time to answer the questionnaire) and a period of at least five minutes in a seated position, all adolescents had their RHR measured three times, and the last two measurements were considered for analysis.

### Outcomes

#### Blood pressure

Blood pressure was measured using the Omron HEM 742 blood pressure monitoring device (Omron, Shanghai, China).^19^ Each adolescent rested in a seated position for at least five minutes, with legs uncrossed. Blood pressure was measured in the right arm placed at the heart level, with an appropriate cuff size. For analysis, the means of the two last measurements were considered, and adolescents were classified as having high blood pressure if they had systolic and/or diastolic blood pressure equal to or greater than the reference sex-, age-, and height-specific 95th percentile.^20^

#### Overweight and abdominal obesity

Height and weight were obtained from adolescents without shoes and coats using a stadiometer and an autonomic scale, respectively. Overweight was determined as proposed by Cole et al.^21^ Waist circumference was obtained in the standing position at the level of the umbilicus using a constant tension tape, and adolescents with waist circumference values above the 80th percentile considering sex and age were considered as having abdominal obesity.^22^

#### Cluster of cardiovascular risk factors

The clustering of cardiovascular risk factors was determined by the sum of abdominal obesity, overweight, and high blood pressure. For analysis, only adolescents with all information were included and the cluster score ranged from 0 to 3.

## Statistical analysis

The three databases were compiled by a single researcher in a data sheet using SPSS/PASW software (IBM Corp, New York, USA) and possible errors were checked by a second independent researcher. Normal distribution was analyzed by graphical analysis (histogram). Continuous variables were summarized as means, with standard deviation and percentiles, whereas categorical variables were summarized as relative frequency. Comparisons of age and RHR between sexes were performed using independent t test, whereas the chi-square test was used for comparing categorical variables (abdominal obesity, overweight, high blood pressure, and Cluster of cardiovascular risk factors).

The sensitivity and specificity of RHR in the detection of cardiovascular risk factors was performed using the receiver operating characteristic (ROC) curve. The association between the adolescents classified with high RHR and the cardiovascular risk factors separated or in the form of a cluster was enhanced by binary logistic regression. The confidence interval adopted was 95%, and the statistical significance was 5%. The statistical packages used were SPSS and Medcalc. A p value < 0.05 was considered statistically significant for all analyses.

## Results

The number of adolescents available, evaluated, and excluded was described previously.^4,17,18^ Thus, a total of 6,794 adolescents (4,040 girls and 2,754 boys) with a mean age of 16.0 ± 1.8 years old were included in the study. Characteristics of adolescents are shown in Table 1. Girls showed higher RHR and abdominal obesity, whereas boys had a higher prevalence of high blood pressure.

**Table 1:** – General characteristics of Brazilian adolescents by sex, in 2011 (n = 6,794)

	Total	Boys (n=2,754)	Girls (n=4,040)	p
Age (years)	16.0 ± 1.8	16.0 ± 1.9	16.0 ± 1.7	0.427^a^
Resting heart rate (bpm)	78.4 ± 12.8	74.4 ± 12.6	81.1 ± 12.2	<0.001^a^
Abdominal obesity (%)	20.7	16.5	23.5	<0.001^b^
Overweight (%)	17.9	18.0	17.8	0.842^ b^
High blood pressure (%)	16.6	24.0	11.6	<0.001^ b ^
**Risk factors (%)**				**<0.001**^**b**^
None	66.1	64.5	67.2	<0.001^ b^
One	17.6	19.0	16.6	
Two	11.3	9.9	12.3	
Three	5.0	6.6	3.9	

*bpm: beats per minute; ^a^: independent t test; ^b^: Chi-square test.*

Smoothed RHR percentiles from adolescents by age and sex are shown in Figure 1 and Table 2. Mean RHR for boys ages 10 to 14 was higher than in boys ages 15 to 19 (80.7 ± 13.0 bpm versus 73.0 ± 12.0 bpm, p < 0.001). Mean RHR for girls ages 10 to 14 was also higher than in girls ages 15 to 19 (83.8 ± 13.0 bpm versus 80.6 ± 12.0 bpm, p < 0.001).

**Figure 1:** – Smoothed resting heart rate for Brazilian adolescent boys and girls (n = 6,794). bpm: beats per minute.

**Table 2:** – Mean ± standard deviation and percentile values for resting heart rate in adolescents ages 10 to 19 years

	Age (years)	Mean ± SD	Resting heart rate percentiles								
			1	2.5	5	10	50	90	95	97.5	99
**Boys (n=2,754)**	10	85.3 ± 14.6	61.4	64.9	68.1	72.1	83.0	108.4	115.2	121.4	129.1
	11	85.7 ± 12.6	58.7	62.1	65.2	69.0	87.5	104.4	110.9	117.0	124.5
	12	84.4 ± 13.6	56.3	59.6	62.6	66.3	83.5	100.7	107.1	113.0	120.3
	13	80.7 ± 11.3	54.3	57.5	60.4	64.0	79.0	97.3	103.5	109.3	116.4
	14	77.8 ± 12.3	52.7	55.8	58.6	62.1	77.8	94.4	100.4	106.0	112.9
	15	76.1 ± 11.6	51.4	54.4	57.1	60.5	75.0	91.8	97.7	103.1	109.8
	16	73.4 ± 12.5	50.4	53.3	56.0	59.2	73.4	89.6	95.3	100.5	107.0
	17	72.1 ± 12.0	49.7	52.5	55.1	58.3	71.0	87.8	93.3	98.3	104.6
	18	70.7 ± 11.5	49.3	52.1	56.6	57.7	70.0	86.4	91.6	96.5	102.5
	19	72.3 ± 10.9	49.8	54.6	56.0	60.0	70.0	86.0	90.0	99.4	111.6
**Girls (n=4,040)**	10	85.9 ± 11.5	61.3	64.5	67.6	71.3	86.0	106.2	112.7	118.8	126.5
	11	86.4 ± 12.4	60,2	63.4	66.4	70.0	84.0	104.2	110.7	116.7	124.1
	12	87.0 ± 13.6	59,3	62.5	65.4	68.9	85.5	102.4	108.7	114.6	121.9
	13	83.4 ± 14.8	58.6	61.7	64.6	68.0	81.0	100.8	106.9	112.6	119.7
	14	82.1 ± 12.0	58.1	61.1	63.9	67.3	81.0	99.2	105.2	110.7	117.6
	15	82.2 ± 12.9	57.7	60.7	63.4	66.8	81.0	97.8	103.6	109.0	115.6
	16	80.1 ± 11.4	57.5	60.4	63.1	66.4	79.0	96.6	102.2	107.3	113.8
	17	80.5 ± 12.1	57.5	60.4	63.0	66.2	79.0	95.5	100.9	105.8	112.0
	18	79.4 ± 11.5	57.7	60.5	63.0	66.2	78.0	94.5	99.7	104.5	110.4
	19	80.1 ± 11.5	54.0	59.3	63.6	67.0	79.0	95.9	102.5	107.0	112.7

*SD: Standard deviation.*

The proposed cutoff points and ROC curve indicators of detection of elevated RHR in both sexes according to age are shown in Table 3. For girls ages 10 to 14, the area under the curve was not significant at the cutoff point found (p > 0.05), whereas, for boys (10 to 14 and 15 to 19 years old) and girls ages 15 to 19, the area under the curve was significant (p < 0.05).

**Table 3:** – Proposed cutoff points and receiver operating characteristic curve indicators of detection of resting heart rate for cardiovascular risk factors in adolescents (n = 6,773)

	Cutoff	AUC	95% CI	p	Sensitivity	95% CI	Specificity	95% CI
Boys 10 – 14 yo	>92	0.632	0.588 – 0.675	0.021	44.44	25.5 – 64.7	81.00	77.1 – 84.5
Boys 15 – 19 yo	>82	0.633	0.612 – 0.653	<0.001	35.96	26.1 – 46.8	82.67	81.0 – 84.2
Girls 10 – 14 yo	>94	0.528	0.486 – 0.570	0.635	32.14	15.9 – 52.4	80.78	77.2 – 84.0
Girls 15 – 19 yo	>82	0.709	0.693 – 0.724	<0.001	75.86	65.5 – 84.4	58.43	56.7 – 60.1

*AUC: area under curve; CI: confidence interval; RHR: resting heart rate; yo: years old.*

Table 4 shows the associations between RHR cutoff points and cardiovascular risk factors. In boys ages 10 to 14, there were associations between elevated RHR and abdominal obesity and overweight. In boys ages 15 to 19, there were associations between elevated RHR and abdominal obesity, overweight, and high blood pressure. In girls ages 15 to 19, as well, elevated RHR was associated with abdominal obesity, overweight, and high blood pressure. In addition, in boys and girls (ages 15 to 19), the RHR cutoff points were independently associated with the cluster of cardiovascular risk factors (Table 5).

**Table 4:** – Crude and adjusted analyses of the association among cutoff points for resting heart rate and cardiovascular risk factors in adolescents

Independent variables	Models	Elevated RHR^a^	
		OR (95% CI)	
**Boys (10 to 14 years old)**			
Abdominal obesity (no = reference)	Crude	2.46 (1.60 – 3.79)	
	Adjusted	2.37 (1.53 – 3.68)	
Overweight (no = reference)	Crude	2.06 (1.38 – 3.10)	
	Adjusted	1.87 (1.23 – 2.83)	
High blood pressure (no = reference)	Crude	1.45 (0.93 – 2.26)	
	Adjusted	1.42 (0.90 – 2.23)	
**Boys (15 to 19 years old)**			
Abdominal obesity (no = reference)	Crude	1.89 (1.27 – 2.82)	
	Adjusted	1.85 (1.24 – 2.76)	
Overweight (no = reference)	Crude	1.88 (1.27 – 2.78)	
	Adjusted	1.89 (1.28 – 2.80)	
High blood pressure (no = reference)	Crude	2.83 (2.01 – 3.98)	
	Adjusted	3.00 (2.12 – 4.23)	
**Girls (15 to 19 years old)**			
Abdominal obesity (no = reference)	Crude	1.22 (1.04 – 1.43)	
	Adjusted	1.26 (1.07 - 1.47)	
Overweight (no = reference)	Crude	1.26 (1.05 – 1.50)	
	Adjusted	1.27 (1.06 – 1.51)	
High blood pressure (no = reference)	Crude	2.86 (2.28 – 3.59)	
	Adjusted	2.82 (2.25 – 3.54)	

*Adjusted for age, period of the day. CI: confidence interval; OR: odds ratio; RHR: resting heart rate. ^a^ Elevated RHR: boys 10 to 14 years old: > 83 bpm; boys 15 to 19 years old: > 92 bpm; girls 10 to 14 years old: unavailable; girls 15 to 19 years old: > 82 bpm.*

**Table 5:** – Adjusted analyses of the association between cutoff points for resting heart rate and clustering of cardiovascular risk factors in adolescents

Risk factors	Elevated RHR^**a**^
	OR (95% CI)
**Boys (10 to 14 years old)**	
None	Reference
One	0.86 (0.50 – 1.49)
Two	2.48 (1.43 – 4.28)
Three	2.33 (1.20 – 4.52)
**Boys (15 to 19 years old)**	
None	Reference
One	3.14 (2.12 – 4.67)
Two	2.22 (1.26 – 3.90)
Three	3.72 (2.12 – 6.54)
**Girls (15 to 19 years old)**	
None	Reference
One	1.33 (1.11 – 1.60)
Two	1.24 (1.01 – 1.54)
Three	3.70 (2.47 – 5.53)

*Adjusted for age, period of the day. CI: confidence interval; OR: odds ratio; RHR: resting heart rate. ^a^ Elevated RHR: boys 10 to 14 years old: > 83 bpm; boys 15 to 19 years old: > 92 bpm; girls 10 to 14 years old: unavailable; girls 15 to 19 years old: > 82 bpm.*

## Discussion

The main findings of the current study were threefold: a) RHR cutoff points have high specificity in detecting the cardiovascular risk in boys ages 10 to 14 and 15 to 19; b) in girls ages 15 to 19, the RHR cutoff points have high sensibility in detecting cardiovascular risk, whereas, in girls ages 10 to 14, the cutoff point could not be established; c) RHR cutoff points identified were independently associated with the cluster of cardiovascular risk factors.

In this study, there was a decline in RHR with increasing age from infancy into adolescence, similarly to others studies.^5,12^ This may be explained by improvements in baroreflex sensitivity and neural function with sexual maturation.^23^ In fact, during maturation there is a progressive increase in parasympathetic cardiac activity related to sympathetic activity,^23^ which supports a lower RHR in late adolescence.^24^

RHR differed between boys and girls ages 15 to 19 (boys: 73.0 ± 12.0 bpm versus girls: 80.6 ± 12.0 bpm), corroborating with previous studies.^4,5^ These differences may be caused by the higher adiposity in girls.^25^ Previous studies have also shown that both cardiorespiratory fitness and physical activity levels are higher in boys than girls,^26^ and these factors are directly related to cardiac autonomic control.^27-29^

Previous studies have investigated RHR reference values in children and adolescents from birth to age 18, including a systematic review involving 143,346 participants, which showed median values of 123 bpm in adolescents ages 12 to 18.^13^ Others studies analyzing large national populations, such as the KiGGS^5^ and NHANES^12^ surveys, observed RHR median values, throughout the age range, varying between 69 and 104 bpm for boys and 74 and 108 bpm for girls. In comparison with these studies, we observed a narrower RHR (median values 70 to 83 bpm for boys and 79 to 86 bpm for girls), which can be explained by the narrow age range.

The main novelty of this study was the identification of cutoff points for RHR in adolescents that were associated with important cardiovascular risk factors for adolescents, namely, abdominal obesity, overweight, and high blood pressure, all of which are independently associated with cardiac autonomic dysfunction,^27,30^ therefore explaining, at least in part, the association with RHR. Likewise, RHR cutoff points were also associated with the cluster of risk factors in a sex-dependent manner, indicating that the accumulation of risk factors leads to more alterations in autonomic function in a dose-dependent manner.

The major strengths of the current study are the relatively large sample size, the coverage of a wide age range, the use of an automated technique for RHR measurements to avoid observer bias, and the fact that data analysis was performed by a single researcher in a blinded fashion. Adolescents with conditions or medication that influencing RHR were excluded. Despite these strengths, this study presents some limitations that need to be considered. First, we had only a single assessment of RHR. We could not determine the Tanner stage. Therefore, future studies should consider the sexual maturation of adolescents. The cross-sectional design of this study is a limitation, as no causality can be inferred, making longitudinal studies necessary to validate the cut points found. Although this is the first study with cutoff points in Brazilian adolescents, we have analyzed few regions in Brazil, making more multicenter studies necessary. Finally, as other potential confounders that may affect RHR (use of tobacco, alcohol consumption, and exercise prior to measurement) were not controlled, their influence cannot be disregarded. Despite that, prior studies have also adopted a similar strategy to the current one.^5,12,13^

## Conclusion

The current study identified > 92 bpm for boys ages 10 to 14, > 82 bpm for boys ages 15 to 19, and > 82 for girls ages 15 to 19 as cutoff points for RHR and demonstrated the association between these cutoff points and the cluster of cardiovascular risk factors. These cutoff points may help physicians and other health professionals to interpret and stage cardiovascular risk, using a simple, easy and low-cost measure.
